# PrEP University: A Multi-Disciplinary University-Based HIV Prevention Education Program

**DOI:** 10.1007/s10900-021-01007-x

**Published:** 2021-06-09

**Authors:** Sophie M. Cannon, Sara Graber, Helen L. King, Marvin Hanashiro, Sarah Averbach, David J. Moore, Jill Blumenthal

**Affiliations:** 1 Department of Medicine, Antiviral Research Center, UCSD Medical Center, 220 Dickinson Street, Suite A, San Diego, CA 92103 USA; 2 Department of Internal Medicine, UT Southwestern, Dallas, TX 75390 USA; 3 Obstetrics, Gynecology and Reproductive Sciences, University of California, San Diego, San Diego, CA 92103 USA; 4 Department of Psychiatry, University of California, San Diego, San Diego, CA 92103 USA

**Keywords:** PrEP, LGBTQ health, Preexposure prophylaxis, LGBTQ medical education, HIV

## Abstract

The introduction of emtricitabine/tenofovir diphosphate (FTC/TDF) as pre-exposure prophylaxis (PrEP) for HIV prevention has raised questions regarding which clinicians will serve as prescribers and how providers will be educated about this HIV prevention strategy. We piloted an HIV Prevention Education Program called PrEP University (PrEP U) to address knowledge gaps in HIV prevention among medical trainees. We examined PrEP awareness and assessed learning as a result of the program, measuring knowledge before and after the lectures with an anonymous 5-question multiple choice test. A total of 198 learners participated in PrEP University, which included 127 first year medical students, and post-graduate trainees in internal medicine (n = 23), family medicine (n = 16), OBGYN (n = 13) and pharmacy (n = 19). Prior to PrEP U, 27% of all participants were not aware of PrEP and an additional 8% were unsure if they had heard of it. Knowledge increased significantly after the education program among trainees in OBGYN (2.3 vs 3.8, p < 0.001), pharmacy (1.4 vs 2.5, p = 0.012) and school of medicine (3.3 vs 4.4, p < 0.001), with a trend seen in family medicine (2.7 vs. 3.7, p = 0.067) and internal medicine (2.7 vs 3.4, p = 0.068). Overall, an HIV Prevention Education Program was successfully administered to nearly 200 participants and resulted in improved knowledge of HIV prevention and PrEP across. Pharmacists and OBGYN physicians are two groups with an expanding role in the use of PrEP. Similar programs at other medical schools should be implemented to ensure that future physicians and pharmacists are comfortable with PrEP prescription.

**Supplementary Information:**

The online version contains supplementary material available at 10.1007/s10900-021-01007-x.

## Introduction

While much progress has been made in the prevention and treatment of HIV/AIDS, there are still roughly 40,000 new HIV cases each year in the United States (US) [1]. In 2012, the Food and Drug Administration (FDA) licensed pre-exposure prophylaxis (PrEP), revolutionizing HIV/AIDS prevention and cultivating a renewed enthusiasm to end the pandemic by 2030 as outlined by the Joint United Nations Programme on HIV and AIDS [2]. This once daily tablet containing a combination of anti-retrovirals emtricitabine and tenofovir disoproxil fumarate (FTC/TDF) or emtricitabine and tenofovir alafenamide (FTC/TAF) has proven to be highly effective as evidenced by multiple clinical trials [3–5]. From 2014 to 2016 the annual number of PrEP users expanded from 13,748 to 78,360, an impressive 470% increase [6]. However, the CDC estimates that roughly 1.2 million U.S. adults could benefit from PrEP [7], highlighting the need for greater HIV prevention efforts.

One common barrier to PrEP usage is experience with and knowledge of PrEP among health care providers. In a survey of primary care doctors in ten US cities with a high incidence of HIV, only 33% of providers had discussed PrEP with patients and only 17% prescribed PrEP [8]. PrEP awareness among providers has increased following the release of the iPrEx study and CDC prescribing guidelines; USA primary care providers are becoming more familiar with PrEP and overall awareness increased from 29% in 2010 to 66% in 2015 [4, 7, 9]. Although PrEP awareness has increased, prescription rates remain low and prescriptions are largely written by a small subset of medical specialties and in specific geographic regions [10].

While awareness may be increasing, attitudes towards PrEP and rates of prescription vary between HIV specialists and generalists. Among primary care providers surveyed in 2015, only one-third had ever prescribed or referred a patient to PrEP services [11]. Unsurprisingly, those with more experience providing care to HIV positive patients were more likely to be prescribers and were less likely to think PrEP usage would result in an increase in risky behaviors [11]. In another study, physicians who perceived PrEP to be ineffective and noted barriers to prescribing were found to have less experience with PrEP [12]. This discrepancy has raised much discussion as to where patients should obtain PrEP and who would be best suited to prescribe this medication.

As highlighted in the “purview paradox,” there is no consensus on which clinicians should be tasked with prescribing PrEP, and neither HIV specialists nor primary care doctors considered PrEP implementation to fall within their clinical domain [13]. HIV providers are proficient in HIV medication management and the ability to assess and counsel around sexual risk behaviors [13], but primary care providers have the ability to reach the largest scope of potential PrEP users as they see HIV-negative patients [14]. In order for PrEP’s potential to be realized, this knowledge base must extend beyond HIV and infectious disease specialists [15] and should include pharmacists, primary care, and Obstetrics and Gynecology (OBGYN) providers. As evidenced by our previous research surveying physicians in California and New York, HIV providers had significantly greater PrEP experience than non-HIV providers, but differences in PrEP prescribing practices were largely dependent on knowledge [16]. The need for educational efforts aimed to bridge this knowledge gap between HIV specialists and other disciplines has been well documented in recent literature [11, 12, 17, 18].

To date, very few PrEP educational programs have been implemented, particularly those specifically written for medical students and residents. In a recent survey of medical students in the Northeastern USA, respondents were less willing to prescribe PrEP to those with multiple partners and inconsistent condom use than to those with a single partner and consistent condom use, further supporting the need for increased education around HIV prevention and PrEP guidelines [19]. Since medical students and residents are taught a relatively standard curriculum, the potential exists to systematically educate learners entering a large range of future specialties, and to use increased knowledge to correct misconceptions and mitigate bias.

In our current study, we piloted a PrEP and HIV prevention curriculum known as PrEP University which was presented to medical students and trainees from disciplines deemed likely to prescribe PrEP at the University of California, San Diego. Through a two-lecture series accompanied by a pre and post-test, we examined PrEP awareness and the ability of a PrEP curriculum to increase PrEP knowledge.

## Methods

### Trainee Groups

Learners who participated in PrEP University included first year medical students in the Practice of Medicine (POM) Course and Internal Medicine (IM), Family Medicine (FM), OBGYN, and Pharmacy residents. These disciplines were selected because they are most likely to discuss or offer PrEP to patients.

### Curriculum Design

PrEP University was developed as a brief HIV prevention education pilot program aimed at teaching physicians- and pharmacists-in-training how to discuss HIV risk, sexual health, and HIV prevention strategies. The overall goal was for medical students and trainees to increase their exposure to and knowledge of talking to patients about sexual health and PrEP. “Trainees” refer to medical students and post-graduate residents. The program provided an individualized 2-part lecture series to different medical disciplines including IM, FM, OBGYN and Pharmacy. The education series was divided into two 60-min lectures given one week apart by two infectious disease specialists with expertise in LGBTQ health. Topics addressed included HIV epidemiology and testing, sexual-history taking, HIV prevention strategies and PrEP education. A modified one-hour lecture was given to first year medical students in their Practice of Medicine (POM) course and focused primarily on the basics of HIV and sexually transmitted infections, sexual history-taking and HIV prevention. Each lecture was adjusted to focus on high-yield points relevant to the particular medical discipline in attendance. For example, the Pharmacy lecture had additional content focused on PrEP’s mechanism of action, whereas the OBGYN lecture included information on PrEP use during pregnancy and breast-feeding. This pilot study took place between September 2016 and June 2017.

### Measures

#### Knowledge-Based Questions

Anonymous pre- and post- education knowledge questions were designed to assess learning as a result of a new PrEP education program. These were distributed at the beginning of the first lecture and the end of the second lecture, with the exception of POM for which only one lecture was given. Pre- and post-tests both included basic demographic questions (age, gender, race, ethnicity, and healthcare role, specialty), in addition to five knowledge-based multiple choice questions with scores ranging 0 (none correct) to 5 (all correct). The knowledge questions included two universal questions given to all sub-specialties, and three individualized questions specific to the specialty receiving the lecture with some overlapping questions (Appendix 1, Table 2). FM and IM received the same set of questions. Pharmacy trainees also received 5 questions, but one question was eliminated for the pre-test and post-test comparison because it did not match. The POM group received a separate set of questions more appropriate for their level of training. With the exception of POM, knowledge scores were compared between pre- and post-test lecture cohorts. Due to the nature and timing of the lectures, individuals were not required to participate in both lecture series to complete these surveys. Baseline knowledge scores of all participants who came to the first lecture were evaluated and compared across specialties. When assessing changes in knowledge, pre- and post-test surveys were matched to a particular individual using age, gender, race/ethnicity and healthcare role to ensure that changes in knowledge were only assessed for participants who attended both lectures.

#### PrEP Awareness

Awareness of PrEP was assessed during both lectures with a question that stated, “Prior to today, had you heard of pre-exposure prophylaxis or PrEP (a biomedical prevention strategy to reduce the risk of HIV in high-risk individuals)?” As above, matching by age, gender, race/ethnicity and healthcare role was used to ensure that a given participant’s answers were not counted twice. Post-test PrEP awareness questions were eliminated if the participant had answered this question during the pre-test prior to the first lecture. Those who had attended only the first or only the second lecture were also counted as unique individuals during data analysis of PrEP awareness.

### Statistics

Descriptive statistics were used for demographics and healthcare role. Paired t-test was used to compare PrEP knowledge scores within IM, FM, Pharmacy, OBGYN and POM groups before and after the lecture series. One-way ANOVA was used to compare the effect of specialty on baseline knowledge scores and PrEP awareness. For baseline knowledge, we ran two models: one comparing all 5 specialties (M1) and one without POM (M2) given their knowledge-based questions were less about PrEP and therefore less comparable to the other 4 groups.

### Ethics

An IRB approval for a waiver of informed consent was obtained by the Institutional Review Board at the University of California, San Diego.

## Results

### Study Population

A total of 198 learners participated in PrEP University, which included 127 first year medical students, 23 IM trainees, 16 FM trainees, 13 OBGYN trainees, and 19 Pharmacy trainees. Participants in PrEP U had a mean age of 25.2 (SD 2.84), 61% were female, 54% were White and 33% were Asian. Seventy percent were medical or pharmacy students and 30% were interns or residents (Table 1).

**Table 1:** Demographics and PrEP awareness among PrEP university participants

	1st year medical students N (%)	Internal medicine N (%)	Family medicine N (%)	OB/GYN N (%)	Pharmacy N (%)	Total N (%)
Total number	127 (64%)	23 (12%)	16 (8%)	13 (7%)	19 (10%)	198 (100%)
Mean age (SD)	23.8 (1.97)	27.13 (2.2)	29.8 (2.2)	28.6 (1.45)	26.21 (1.55)	25.2 (2.84)
Gender						
Male	54 (27%)	11 (6%)	7 (4%)	1 (< 0.5%)	4 (2%)	77 (39%)
Female	73 (37%)	11 (6%)	9 (5%)	12 (6%)	15 (8%)	120 (61%)
No report/NA	0	1 (< 0.5%)	0	0	0	1 (< 0.5%)
Race						
White	69 (35%)	10 (5%)	11 (6%)	11 (6%)	6 (3%)	107 (54%)
Black	3 (2%)	0	0	0	1 (< 0.5%)	4 (2%)
Asian	43 (22%)	10 (5%)	2 (1%)	2 (1%)	8 (4%)	65 (33%)
Other	1 (< 0.5%)	2 (1%)	1 (< 0.5%)	0	3 (2%)	7 (4%)
No report/NA	11 (6%)	1 (< 0.5%)	2 (1%)	0	1 (< 0.5%)	15 (8%)
Ethnicity						
Latinx	14 (7%)	0	0	2 (1%)	2 (1%)	18 (9%)
Not Latinx	102 (52%)	21 (11%)	14 (7%)	10 (5%)	16 (8%)	163 (82%)
No report/NA	11 (6%)	2 (1%)	2 (1%)	1 (< 0.5%)	1 (< 0.5%)	17 (9%)
Healthcare role						
Medical student	127 (64%)	8 (4%)	0	0	0	135 (68%)
Pharmacy student	0	0	0	0	4 (2%)	4 (2%)
Intern/resident	0	15 (8%)	16 (8%)	13 (7%)	15 (8%)	59 (30%)
Aware of PrEP						
Yes	67 (34%)	19 (10%)	16 (8%)	8 (4%)	19 (10%)	129 (65%)
No	46 (23%)	3 (2%)	0	4 (2%)	0	53 (27%)
Not sure	14 (7%)	1 (< 0.5%)	0	1 (< 0.5%)	0	16 (8%)

*SD* standard deviation, *NA* not available

### PrEP Awareness

Prior to PrEP U, 27% of all participants were not aware of PrEP and an additional 8% were unsure if they had heard of it (Table 1). Forty-seven percent of medical students, 38% of OBGYN trainees, and 17% of IM trainees were not aware of or unsure if they had heard of PrEP as opposed to all of those surveyed in FM and Pharmacy who were aware of PrEP prior to completing the lecture series. There was a statistically significant effect of specialty on PrEP awareness at the [F(4, 193) = 17.44, p < 0.001]. Post hoc comparisons using Tukey HSD test indicated that PrEP awareness for POM was significantly lower than IM, FM and Pharmacy (all p < 0.001) but not for OBGYN (p = 0.29). There were no other differences found between groups (Table 2).

**Table 2:** Distribution of questions among specialties

Question	IM	FM	OBGYN	Pharmacy	POM
How many people are living with HIV worldwide?	**X**	**X**	**X**	**X**	**X**
Which patient is considered at substantial risk for HIV infection by the CDC definition?	**X**	**X**	**X**		**X**
Which is NOT a proven effective method of preventing HIV?	**X**	**X**	**X**		**X**
Which of the following is NOT true about the fourth generation HIV test?	**X**	**X**	**X**	**X**	
Which study showed efficacy in reducing HIV infection using oral PrEP in MSM and transgender women?	**X**	**X**		**X**	
How many days does it take to achieve maximum protection in the anal mucosa when starting Truvada as PrEP?				**X**	
Rates of diagnoses of HIV infection in the US are highest among which age group?					**X**
The CDC provides 5 categories (the 5 P’s) for sexual history questions when interviewing a patient. Which is NOT one of the 5 P’s?					**X**
Which study showed efficacy in reducing HIV infection using microbicides?			**X**		

### PrEP Knowledge

Using M1, there was a statistically significant effect of specialty on PrEP baseline knowledge at the p < 0.05 level [F(4, 177) = 15.78, p < 0.001]. Post hoc comparisons using Tukey HSD test indicated that PrEP knowledge for Pharmacy was significantly lower than IM [(Mean Difference (MD) = − 1.19, p = 0.005] and POM (MD = − 1.86, p < 0.001) with a trend for FM (MD = − 1.12, p = 0.062) but no difference compared to OBGYN (MD = − 0.65, p = 0.43). In addition, PrEP knowledge for POM was significantly greater than Pharmacy (MD = 1.86, p < 0.001) and OBGYN (MD = 1.21, p = 0.002) with a trend for IM (MD = 0.67, p = 0.057) but no difference compared to FM (MD = 0.74, p = 0.20). Using M2, there remained a statistically significant effect of specialty on PrEP baseline knowledge at the p < 0.05 level [F(3, 51) = 4.64, p = 0.006] with post hoc comparisons using Tukey HSD test indicating that PrEP knowledge for Pharmacy was significantly lower than IM (MD = − 1.19, p = 0.005) and FM (MD = − 1.12, p = 0.048) but no difference compared to OBGYN (MD = − 0.65, p = 0.36).

**Fig. 1:**
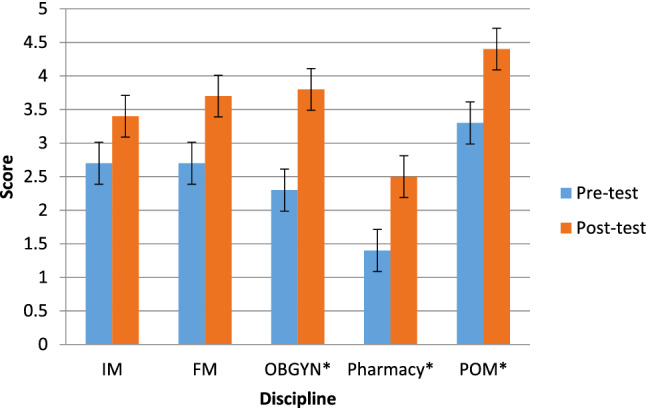
Changes in Knowledge Scores Across Disciplines. Knowledge scores from pre-test (blue) and post-test (orange) across disciplines. IM (2.7 vs 3.4, p = 0.068), OBGYN (2.3 vs 3.8, p < 0.001), Pharmacy (1.4 vs 2.5, p = 0.012) and POM (3.3 vs 4.4, p < 0.001), FM (2.7 vs. 3.7, p = 0.067). *Statistically significant difference from pre-test to post-test (p < 0.05)

Knowledge scores increased across specialties after participating in the education program. Significantly higher post-test scores, representing an increase in knowledge, were observed for OBGYN (2.3 vs 3.8/5; t = − 7.94, p < 0.001), Pharmacy (1.4 vs 2.5/4; t = − 3.026, p = 0.012) and POM (3.3 vs 4.4/5; t = − 12.27, p < 0.001). Although not statistically significant, trends were noted for IM (2.7 vs 3.4/5; t = − 1.98 p = 0.068) and FM (2.7 vs 3.7 / 5; t = − 2.12, p = 0.067) (Fig. 1).

## Discussion

### Main Findings

A brief HIV prevention education program was successfully created and presented to nearly 200 participants. Our goal for this pilot study was to increase PrEP knowledge so that trainees across a variety of specialties would increase their knowledge of and exposure to PrEP, in addition to patients at risk for HIV who would benefit from receiving this medication. HIV prevention was also part of the curriculum, as trainees must have a foundation in both HIV and its prevention in order to comprehensively understand the need and use for PrEP. This curriculum resulted in improved knowledge of HIV prevention and PrEP across disciplines (with a trend noted for IM and FM) and among first-year medical students, with specialty-specific findings related to Pharmacy and OBGYN trainees.

Overall, all trainee groups had relatively good awareness of PrEP with the exception of first year medical students. This deficit in medical student awareness of PrEP may simply reflect level of training as it pertains to exposure to PrEP, which has been similarly found in other studies [20]. Post-graduate trainees likely learned about PrEP in medical school or over the course of their training, with medical students actively learning about PrEP in curricula such as the one offered. Research suggests that there is indeed an interest and need to educate medical students about HIV risk and PrEP [21].

Despite having high levels of PrEP awareness, pharmacy trainees had lower baseline knowledge scores compared to most other specialties. Pharmacists are a unique group with an expanding role in the use of PrEP and are an excellent resource for improving patient understanding, promoting medication adherence, and providing counseling to enhance PrEP use [22]. Patients who obtain outpatient prescriptions for medications related to STD treatment, oral contraceptives, and over-the-counter items related to sexual activity often interact with pharmacists and may benefit from such sexual health counseling and PrEP education [23]. However, there are significant knowledge gaps amongst pharmacists. In a 2016 PrEP awareness study conducted in Iowa and Nebraska, less than half of community pharmacists were familiar with the use of PrEP or the guidelines for its use. Further, older and more experienced pharmacists were less familiar with PrEP, perhaps reflecting the recent inclusion of PrEP content in pharmacy school curricula [17, 24]. In a similar survey conducted in Minnesota, 70% of pharmacists reported they had never received an inquiry about PrEP from patients and only 33% reported dispensing PrEP, with the most common concern being the identification of appropriate candidates for PrEP [17, 23]. These knowledge barriers to dispensing PrEP can be addressed and mitigated by educational initiatives in pharmacy curricula such as PrEP University.

Most states allow pharmacists to initiate, discontinue, and monitor medications under a Collaborative Drug Therapy Agreement, which presents a unique opportunity for pharmacists to prescribe PrEP directly to patients. There are clinics in several states trialing pharmacist-delivered PrEP services [22, 25, 26]. As discussed above, there remains no consensus on which clinicians should be tasked with prescribing PrEP [13]. Our study sheds light on the relative high awareness, but low knowledge, of specifics related to PrEP in our pharmacy trainees. Clearly, pharmacists are a key provider group for which targeted PrEP education is appropriate and necessary as PrEP prescription by pharmacists becomes more widespread across the USA. As such, educational initiatives are imperative in increasing knowledge of PrEP for pharmacists in training. While not addressed in our pilot study, intent to prescribe and comfort with prescribing are important aspects of pharmacy-lead PrEP initiatives that warrant further investigation.

In addition to the expanding role of pharmacists, the front line of preventative sexual health falls not only on HIV specialists and primary care doctors but also on OBGYNs. OBGYNs provide 44% of preventative care to nonpregnant women in the United States, including sexual and reproductive healthcare, and are thus likely to treat patients who may benefit from PrEP [24]. There is evidence to suggest that PrEP use during pregnancy and lactation is safe and effective, which would be important to communicate during the pre- and peripartum period. OBGYNs as well as family planning providers should play a critical role in discussing and prescribing PrEP both in the preventative setting and during pregnancy [24, 27]. Yet despite guidelines from the American College of Obstetricians and Gynecologists (ACOG) regarding PrEP administration surrounding pregnancy [28], to our knowledge there is no current data that captures the PrEP prescribing practices of OBGYN providers. Studies assessing PrEP awareness and knowledge of OBGYNs are also lacking, and our study represents one of the first to investigate this [29]. While it warrants further study, the lack of published data could suggest a lack of prescribing on the part of OBGYN providers, and thus denotes a key group of physicians who might benefit from increased education during pivotal parts of their medical school and residency training. In our study, we found that while many OBGYN trainees had heard of PrEP, knowledge was poor but scores increased with the implementation of our lecture series. As research suggests that increased provider knowledge of PrEP is associated with higher rates of prescription and future intent to prescribe, augmenting education represents an important first step in implementing meaningful change [16].

However, while studies suggest knowledge may increase intent to prescribe, other barriers exist. As is well documented in recent literature, both provider and patient stigma may impact PrEP’s utilization [30, 31]. Though PrEP stigma is often experienced by potential users as it relates to the stigma of HIV, provider bias plays a significant role, with authors noting, “PrEP users are stigmatized because they are seen as *wanting* to engage in behavior that previously would have put them at risk for HIV infection, even if that risk has been eliminated by PrEP use” [31]. The availability of PrEP for patients based on high risk sexual behavior also contributes to provider stigma, corresponding with negative perceptions of patients who might be eligible for PrEP and reluctance to prescribe to patients deemed not risky enough [11, 31–33]. As indicated above, in a recent survey of medical students in the Northeastern US, respondents were less willing to prescribe PrEP to those with multiple partners and inconsistent condom use than to those with a single partner and consistent condom use [19], highlighting the need for PrEP education. While not explicitly addressed in our model, educating trainees while also teaching about stigma, bias and normalizing alternate sex practices is important to consider in future iterations of this pilot study.

### Limitations

Our study is not without limitations. While nearly 200 participants completed the series, they were not spread evenly among the disciplines, which limited our ability to perform analyses based on specialty or basic demographic information. Due to the nature of the lecture series and how the pre- and post-test surveys were given and collected, individuals did not need to attend both lectures in order to participate in PrEP University. However, this issue was addressed in several ways: (1) we matched individuals within specialties to compare pre- and post-knowledge scores and (2) we removed repeat participants via matching to accurately assess unique participant demographic information as well as reported PrEP awareness and baseline knowledge. In addition, the demographics of our study participants were largely homogenous with respect to racial and ethnic diversity, though similar when compared to USA physician workforce data [34]. However, this limits our ability to generalize these results to more heterogeneous groups of healthcare providers both nationally and internationally. Importantly, we acknowledge the low number of Black/African American and Hispanic/Latinx participants in our study and the ongoing need for increased representation among providers. Further, while not addressed in our current study, comfort with prescribing, intent to prescribe and assessment of stigma and bias after receiving such a lecture series would be important data in future PrEP-education based studies. Finally, while all disciplines were given 5 questions to assess knowledge, not all of the questions between groups were identical, and one question from the pharmacy set was eliminated due to a mismatch in one pre- and post-test question, making cross comparisons more difficult.

## Conclusion

Given the success of this brief HIV prevention education program, similar programs at other university-based medical schools around the country should be implemented to ensure that future practicing physicians and pharmacists understand the use of PrEP, particularly in regions of high HIV incidence. We recognize that there may be barriers to implementation of such a lecture series in teaching environments that have limited resources or knowledge experts to teach about PrEP. However, the creation of a standardized PrEP curriculum that could be recorded, offered virtually/online, and distributed across a variety of MedEd teaching environments is an exciting possibility.

## Supplementary Information

Below is the link to the electronic supplementary material.

Supplementary file1 (DOCX 15 kb)

## Data Availability

Data available on request.
