# Supplementary material for: PrEP University: A Multi-Disciplinary University-Based HIV Prevention Education Program
Source: J Community Health. 2021 Jun 9;46(6):1213–20. doi: 10.1007/s10900-021-01007-x (PMC8595182; doi:10.1007/s10900-021-01007-x)
Supplement: Supplementary file 1 — Supplementary file1 (DOCX 15 kb) [file 10900_2021_1007_MOESM1_ESM.docx]

Appendix 1: Knowledge-based questions completed by Internal Medicine, Family Medicine, OBGYN, Pharmacy, and/or Practice of Medicine

Which of the following is NOT true about the fourth generation HIV test?

1. It is a combined antigen and antibody test
2. Its use decreases the time in the window period
3. It has nearly 100% sensitivity and specificity in chronic HIV infection
4. It detects the HIV-1 p24 antigen
5. **It is the last step in the CDC recommended HIV testing algorithm**

How many people are living with HIV worldwide?

1. 198 thousand
2. 7.4 million
3. 15.7 million
4. **37 million**
5. 152 million

Which study showed efficacy in reducing HIV infection using oral PrEP in MSM and transgender women?

1. CAPRISA
2. VOICE
3. DISCOVER
4. **iPrEx**
5. FEM PrEP

How many days does it take to achieve maximum protection in the anal mucosa when starting Truvada as PrEP?

1. 24 hours
2. 3 days
3. **7 days**
4. 30 days
5. 45 days

Which patient is considered at substantial risk for HIV infection by the CDC definition?

- 1. Anyone in an ongoing sexual relationship with an HIV-positive partner
  2. A woman who has had anal sex with a man who injects drugs without a condom in the past 6 months
  3. A woman who has oral sex only with a single partner whose HIV-status is unknown
  4. **A and B**
  5. All of the above

Which is NOT a proven effective method of preventing HIV?

1. An HIV-uninfected patient taking once a day HIV medication
2. An HIV-infected patient takes HIV medications every day, which prevents transmission of the virus to HIV-uninfected partners
3. Condoms
4. **Pulling out or removing the penis prior to ejaculation**
5. All of the above are effective methods of preventing HIV

Which study showed efficacy in reducing HIV infection using microbicides?

- 1. **CAPRISA**
  2. VOICE
  3. MTN 003
  4. FACTS 001
  5. FEM PrEP

How many people are living with HIV worldwide?

1. 198 thousand
2. 7.4 million
3. 15.7 million
4. **37 million**
5. 152 million

Rates of diagnoses of HIV infection in the US are highest among which age group?

1. 13-24 years old
2. **25-34 years old**
3. 35-44 years old
4. 45-54 years old
5. ≥ 55 years old

The CDC provides 5 categories (the 5 P’s) for sexual history questions when interviewing a patient. Which is NOT one of the 5 P’s?

1. Partners
2. **Privacy**
3. Past History of Sexually Transmitted Infections
4. Practices
5. Pregnancy Plans
